# Les accessoires infantiles en métal: quand un bijou devient un réel risque de perte oculaire

**DOI:** 10.11604/pamj.2019.33.255.19678

**Published:** 2019-07-26

**Authors:** Maryama El Kaddoumi, Ouafa Cherkaoui

**Affiliations:** 1 Service d'Ophtalmologie A, Université Mohamed V, CHU Ibn Sina, Rabat, Maroc

**Keywords:** Urgences ophtalmologiques, perte oculaire, accessoires infantiles en métal, Ophthalmic emergencies, eye loss, metal accessories for children

## Abstract

This study involved a 10-year old female patient admitted to the Ophthalmic Emergency Department with domestic trauma caused by her metal jewelry hung to the conjunctiva and the eyelid. Ophthalmic examination was strictly normal with the exception of the foreign body remarkably visible to the naked eye. The ablation of the foreign body resulted in localized subconjunctival haemorrhage without palpebral lesions, lesions of the right lateral muscle, conjunctival or scleral lacerations. The patient underwent treatment with artificial tears and antibiotics with good evolution. Tetanus vaccination was updated. It is essential to pay attention to metal accessories for children but also to metal accessories for the entire population.

## Image en médecine

Il s'agit d'une petite fille de 10 ans, admise aux Urgences Ophtalmologiques suite à un traumatisme domestique par son bijou en métal, accroché à la conjonctive et à paupière. L'examen clinique ophtalmologique était strictement normal à l'exception du corps étranger remarquablement visible à l'œil nu, l'ablation du corps étranger à laisser place à une hémorragie sous conjonctivale localisée sans lésions palpébrales ou du muscle droit latéral ni de lacération conjonctivale ou sclérale. La patiente a bénéficié d'un traitement par larmes artificielles et antibiotiques avec bonne évolution. La vaccination antitétanique était à jour. Attention aux accessoires en métal chez les enfants mais aussi chez toute la population.

**Figure 1:**
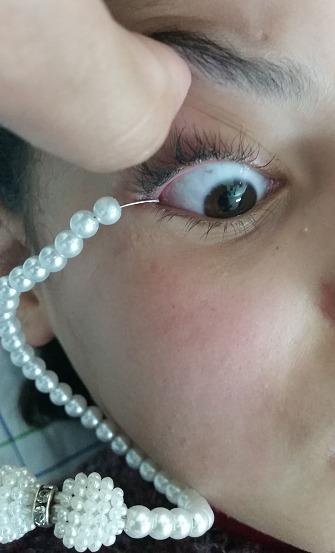
Accessoires infantiles en métal

